# Notes and News

**Published:** 1897-09-18

**Authors:** 


### Notes and News.

(Collected and Communicated.)

The *Medical Record* of New York, edited by the well-known Dr. George F. Shrady, comments on the action of the British Medical Association in expelling Dr. Leith Napier and Dr. Ramsay Smith in the following terms: "The scramble for hospital appointments which have been vacated by colleagues under pressure from their lay masters is not regarded with pleasure by disinterested onlookers, but if every man who had gleefully taken a brother's place were expelled from his medical society there would be a sad thinning out indeed."

Homœopathy seems to be a perennial cause of disputation in the United States. The directors of the Hahnemann Hospital College in San Francisco have now petitioned for affiliation with the University of California, and a counter petition has been presented by the Medical Department of the University. No doubt it is a very emasculated form of homœopathy which is so largely current in the States; but that is all the more reason why it should be refused recognition. Anything good which may have sprung from Hahnemann's crazy dogma, such, for example, as the enlarged opportunities for studying the natural history of disease, has long since been utilised to the full by medical men, and what has not been so utilised is mere bunkum and pretence.

At the last quarterly court of the Radcliffe Infirmary, Oxford, the question arose again in respect to the expenditure in alcohol. The expenditure under this head was £24 larger than in the corresponding quarter of the previous year. This item has always been an exceptionally large one; it is entirely in the hands of the medical staff, one of whom explained that the unusual number of severe cases caused the increase referred to. At the same meeting the new treasurer, Sir John Conroy, suggested various improvements within the institution, and we hope that he will succeed in diffusing sufficient life into the management to bring the infirmary up to the modern standard of completeness and efficiency.

The Middlesex Hospital has received a further sum of £2,000, being part of a sixth share of the residuary estate of the late Mr. David Brandon.

The American Victoria Jubilee Fund has made a gift to the Seamen's Hospital Society of £1,000, to endow in perpetuity a bed in their branch hospital situated in the Royal Victoria and Albert Docks.

Contributions to the Prince of Wales's Hospital Fund have been received from the Barberton (South Africa) Queen's Jubilee Celebration Committee, £65; and £16 10s. from Messrs. Dorgen and Co., being a subscription of 10s. per case on their champagne sold in this country.

The last report of the Stockton and Thornaby Hospital offers a striking contrast to affairs at the Radcliffe Infirmary, Oxford. The consumption of alcohol at the former institution has been reduced during the last fifteen years from 1·08d. to ·35d. per patient per annum. Further, in the same period the cost per patient has been considerably reduced.

We have not heard very much of the effect of Li Hung Chang's visit to England upon the institutions of his country. Now we learn that it has already resulted in a wider scope being provided for the employment and education of women. In medicine he has given especial encouragement by employing the first lady practitioner in China to be physician to the women of his household. Two Chinese lady doctors are to act as delegates at the Women's Congress in London in 1898.

A writer in a South African paper, speaking from experience, warns consumptives who have not at least £200 to £300 to spend, to avoid the Karoo. With a sufficient income at command, he points out that the conditions are far more favourable than at the European resorts for consumptives, and this testimony is well borne out. At the same time, the custom of impecunious invalids in the last stage of consumption flocking to South Africa is one that should be resisted, as the experienced warn such travellers that no benefit is to be derived under the circumstances, and the misery of a friendless death-bed in comfortless circumstances too frequently is the result.

In connection with the Medical Congress at Moscow a local photographer announced in a circular that he intended to prepare an album of photographs of the members to be presented to the Czar and other distinguished patrons of the Congress. He addressed his English readers thus: "S take the liberty of inviting you dear zir, to favour me with your visit to my artistical installation to the effect of being graciously photographed, or to lit me kindly hane a portrait of yours whereof S could take a copy. Price of album be Rs. 5. My address is noted at the head of this. The installation is oper: the days of Congress at 8 o'clock in the morning untill evening; all other days from 10 to 5 in the afternoon." The announcement was headed with the striking word, "Notire."

